# Virus-Like Particles Containing the E2 Core Domain of Hepatitis C Virus Generate Broadly Neutralizing Antibodies in Guinea Pigs

**DOI:** 10.1128/jvi.01675-21

**Published:** 2022-03-09

**Authors:** Joey McGregor, Joshua M. Hardy, Chan-Sien Lay, Irene Boo, Michael Piontek, Manfred Suckow, Fasséli Coulibaly, Pantelis Poumbourios, Rob J. Center, Heidi E. Drummer

**Affiliations:** a Burnet Institute, Melbourne, Australia; b Department of Microbiology and Immunology at The Peter Doherty Institute for Infection and Immunity, University of Melbourne, Melbourne, Australia; c Infection Program, Biomedicine Discovery Institute, Monash University, Clayton, Australia; d Department of Biochemistry and Molecular Biology, Monash University, Clayton, Australia; e ARTES Biotechnology GmBH, Langenfeld, Germany; f Department of Microbiology, Monash University, Clayton, Australia; University of Southern California

**Keywords:** hepatitis C virus, vaccine, glycoprotein E2, virus-like particle

## Abstract

A vaccine to prevent hepatitis C virus (HCV) infection is urgently needed for use alongside direct-acting antiviral drugs to achieve elimination targets. We have previously shown that a soluble recombinant form of the glycoprotein E2 ectodomain (residues 384 to 661) that lacks three variable regions (Δ123) is able to elicit a higher titer of broadly neutralizing antibodies (bNAbs) than the parental form (receptor-binding domain [RBD]). In this study, we engineered a viral nanoparticle that displays HCV glycoprotein E2 on a duck hepatitis B virus (DHBV) small surface antigen (S) scaffold. Four variants of E2-S virus-like particles (VLPs) were constructed: Δ123-S, RBD-S, Δ123A7-S, and RBDA7-S; in the last two, 7 cysteines were replaced with alanines. While all four E2-S variant VLPs display E2 as a surface antigen, the Δ123A7-S and RBDA7-S VLPs were the most efficiently secreted from transfected mammalian cells and displayed epitopes recognized by cross-genotype broadly neutralizing monoclonal antibodies (bNMAbs). Both Δ123A7-S and RBDA7-S VLPs were immunogenic in guinea pigs, generating high titers of antibodies reactive to native E2 and able to prevent the interaction between E2 and the cellular receptor CD81. Four out of eight animals immunized with Δ123A7-S elicited neutralizing antibodies (NAbs), with three of those animals generating bNAbs against 7 genotypes. Immune serum generated by animals with NAbs mapped to major neutralization epitopes located at residues 412 to 420 (epitope I) and antigenic region 3. VLPs that display E2 glycoproteins represent a promising vaccine platform for HCV and could be adapted to large-scale manufacturing in yeast systems.

**IMPORTANCE** There is currently no vaccine to prevent hepatitis C virus infection, which affects more than 71 million people globally and is a leading cause of progressive liver disease, including cirrhosis and cancer. Broadly neutralizing antibodies that recognize the E2 envelope glycoprotein can protect against heterologous viral infection and correlate with viral clearance in humans. However, broadly neutralizing antibodies are difficult to generate due to conformational flexibility of the E2 protein and epitope occlusion. Here, we show that a VLP vaccine using the duck hepatitis B virus S antigen fused to HCV glycoprotein E2 assembles into virus-like particles that display epitopes recognized by broadly neutralizing antibodies and elicit such antibodies in guinea pigs. This platform represents a novel HCV vaccine candidate amenable to large-scale manufacture at low cost.

## INTRODUCTION

The treatment of hepatitis C virus (HCV) infection has significantly improved in recent years due to the development of highly effective direct-acting antivirals that cure HCV in >95% of cases (1–3). However, direct-acting antivirals are unlikely to achieve the elimination of HCV in most countries globally because of difficulties in accessing treatment and high rates of reinfection (1, 4). In addition, it is estimated that only 50% of people living with HCV have been diagnosed and are aware of their infection (5). Essential to the global elimination of HCV is a safe, low-cost prophylactic vaccine to prevent infection of HCV-naive people and to prevent reinfection of those already cured of HCV (6).

HCV is an enveloped RNA virus of the *Flaviviridae* family and exists as 8 highly divergent genotypes and over 67 subtypes, which differ by at least 30% and 25%, respectively, at the nucleotide level (7–9). This high degree of sequence diversity presents a challenge to development of a vaccine effective against all circulating strains. To date, the majority of successful viral vaccines have been based on the production of neutralizing antibodies (NAbs) that target viral surface proteins (10). For HCV, evidence suggests that both broadly neutralizing antibody (bNAb) responses toward the HCV envelope proteins and a multispecific cellular immune response including both CD4^+^ and CD8^+^ T cells contribute to protection from chronic HCV infection (11–14). Two surface glycoproteins decorate the viral envelope, with the larger surface glycoprotein, E2, containing most known neutralization domains. Located within E2 are three hypervariable regions: HVR1 (residues 384 to 410), HVR2 (residues 460 to 485), and VR3, or the intergenic variable region (igVR) (residues 570 to 580) (H77c amino acid numbering used here and throughout; GenBank accession number AF009606). These regions have been found to contribute to epitope shielding and immune evasion (15–17). We have previously shown that by removing the variable regions of E2 and/or replacing them with flexible linker sequences, a recombinant soluble protein (Δ123) can be expressed that retains a majority of E2-directed NAb epitopes and CD81 binding (18). In experimental animals, a high-molecular-weight (HMW) soluble form of Δ123 was able to elicit bNAbs (19), in part because it occludes nonneutralizing epitopes, favoring the generation of antibodies to the neutralizing face of E2. Recombinant protein-based vaccines are expensive and difficult to manufacture, and this may ultimately restrict vaccine access in low- and middle-income countries.

One potentially low-cost vaccine platform is the production of virus-like particles (VLPs). Virus-like particles closely mimic the size and properties of native virions but with the advantage of being noninfectious, as they contain no genetic material (20). Antigens are displayed on the VLP surface in a multivalent array and can promote efficient cross-linking of B cell receptors to induce strong antibody responses (20–22). Therefore, the presentation of B cell epitopes on a VLP could be an effective vaccine strategy. Licensed prophylactic VLP vaccines such as Engerix b (GSK) for hepatitis B virus (HBV) and Gardasil (Merck) for human papillomavirus demonstrate that VLPs can be a highly effective and safe vaccine platform.

In this study, we engineered a nanoparticle VLP-based HCV vaccine candidate using the S antigen of duck hepatitis B virus (DHBV) to form VLPs that display the HCV E2 glycoprotein as a surface antigen. DHBV is closely related to human HBV in regard to genome organization and mode of replication (23). Naturally occurring virions of DHBV consist of the small (S) and large (L) surface antigens. The DHBV S antigen, like its HBV counterpart, has the capacity to self-assemble into VLPs with a diameter of ∼45 nm (24) and can tolerate insertions of foreign sequences at either the N or C terminus (25). Alignment of the HBV and DHBV S protein sequences shows that their identity and similarity are 25.3% and 32.7%, respectively (Needleman-Wunsh algorithm [EMBL-EBI]), indicating that they are highly divergent. The use of DHBV S antigen rather than HBV S as an antigenic carrier is therefore less likely to reduce vaccine potency as a result of preexisting immunity in the population to HBV S antigen.

We genetically fused the HCV E2 ectodomain to the N terminus of the DHBV S antigen and demonstrated the self-assembly of VLPs in a mammalian expression system. The E2 antigen on the VLPs was recognized by a diverse panel of linear and conformation-dependent broadly neutralizing monoclonal antibodies (bNMAbs). Immune sera were raised in guinea pigs that, for 3/8 of the immunized animals, had the capacity to neutralize 7 HCV genotypes *in vitro*. This indicates that E2-S VLPs are a novel and promising HCV vaccine candidate.

## RESULTS

### Production of E2-S chimeric VLPs.

**FIG 1:**
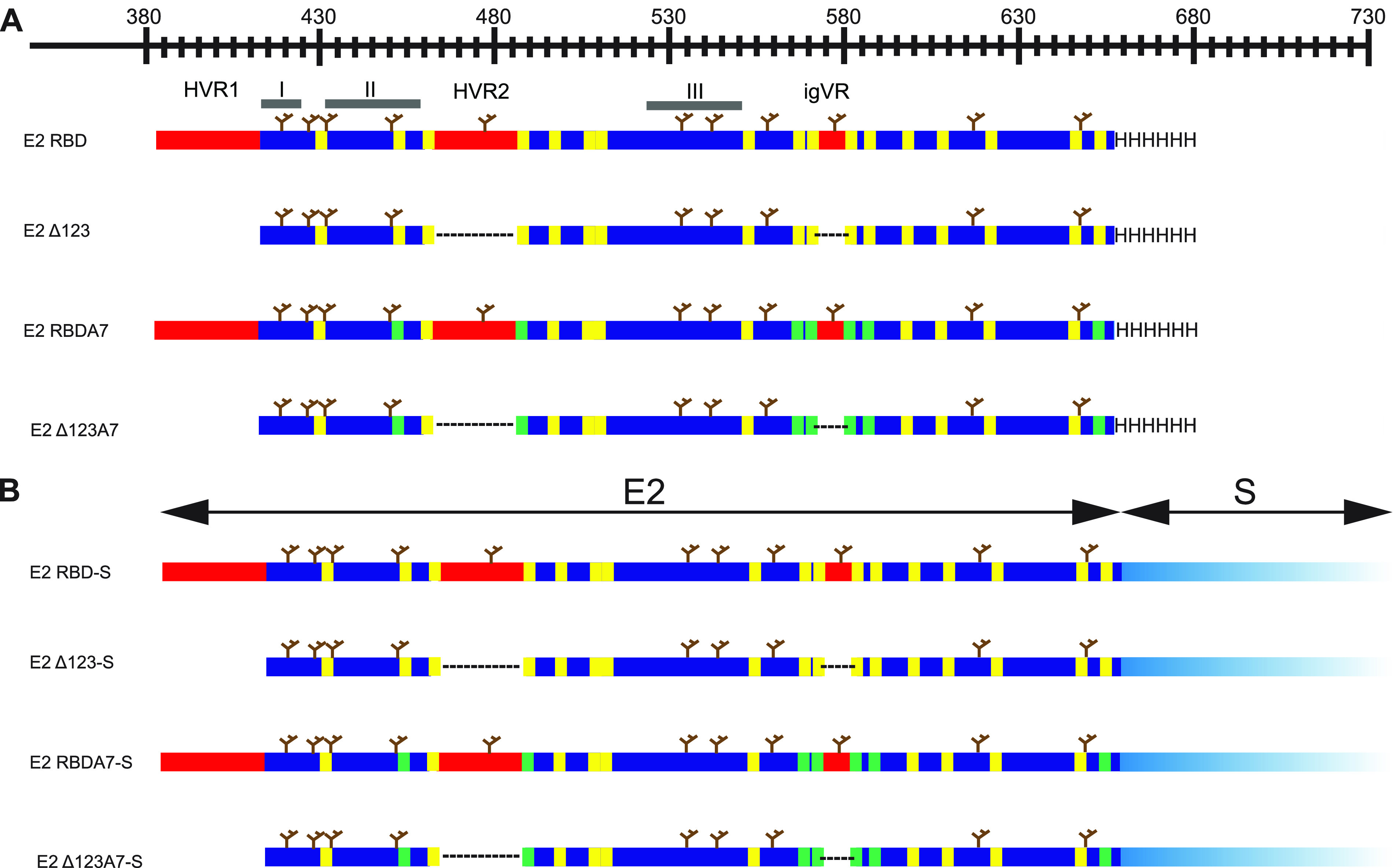
Schematic representation of constructs used in this study. (A) Soluble proteins RBD, Δ123, RBDA7, and Δ123A7. Scale is a representation of the amino acid numbering according to the H77c prototype. Variable regions (red), variable regions replaced by GSSG linkers (dotted lines), epitope I, II, and III regions (thick gray lines), N-linked glycans (brown trees), cysteine residues (yellow lines), Cys-Ala mutations (green lines), and 6×His tag (HHHHHH) are indicated. (B) E2-S constructs where E2 is fused at the C terminus to the N terminus of S (light blue). Regions within the E2 area are as described for panel A.

**FIG 2:**
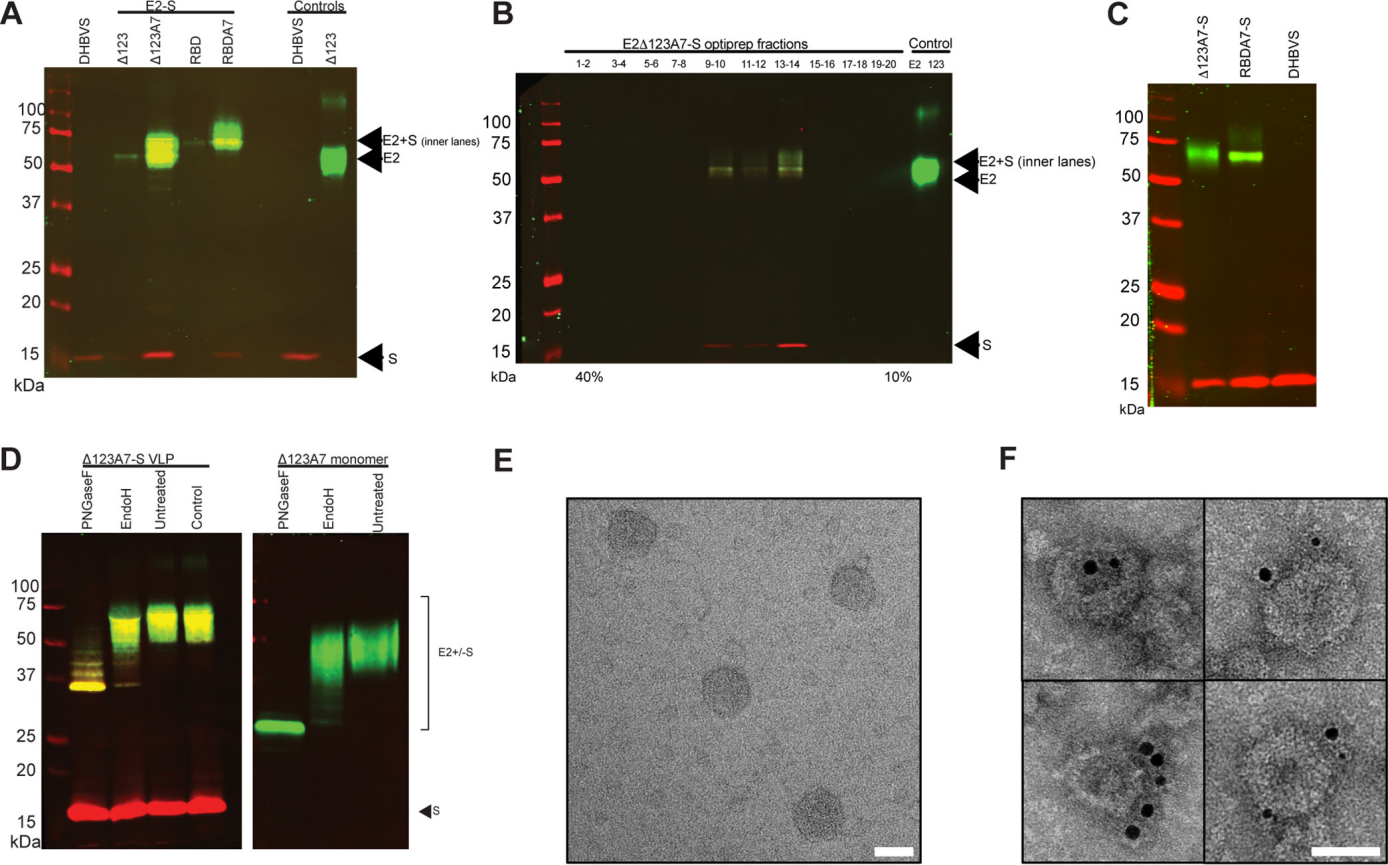
Western blot analysis of E2-DHBVS VLPs showing the expression of DHBVS alone and 4 variants of E2-S VLPs. HEK293T cells were cotransfected with E2-S and DHBVS and VLPs purified by ultracentrifugation through a 20% sucrose cushion. Reducing SDS-PAGE was performed, followed by Western blotting with anti-E2 bNMAb HCV1 (green) and anti-S MAb 7C12 (red). Purified soluble protein Δ123 and DHBVS were included as controls. (B) Immunoblot of Δ123A7-VLPs sedimented through an iodixanol gradient. VLP-containing fractions were resolved by reducing SDS-PAGE followed by Western blotting with anti-E2 bNMAb HCV1 (green) and anti-S MAb 7C12 (red). (C) Immunoblot of VLPs after E2-S and DHBVS-containing fractions were pooled, pelleted, and resuspended in PBS. (D) Deglycosylation of Δ123A7-S or Δ123A7 soluble protein with either PNGase F or Endo-H. Untreated, no enzyme; control, no enzyme or DTT. Analysis was done as described for panel A. (E) Transmission electron microscopy of purified E2Δ123A7-S VLPs negatively stained with 1% (wt/vol) uranyl acetate. (F) Immunoelectron microscopy of purified E2Δ123A7-S VLPs incubated with human MAb HC84.27 (anti-E2), mouse MAb 7C12 (anti-S), 5-nm gold-conjugated anti-human immunoglobulin, and 10-nm gold-conjugated anti-mouse immunoglobulin, followed by staining with 2% (wt/vol) uranyl acetate. Scale bars (E and F) = 50 nm.

E2-S chimeras were constructed by fusing the C termini of the H77c receptor-binding domain (RBD), Δ123, RBDA7, and Δ123A7 to the N terminus of DHBV S antigen to generate RBD-S, Δ123-S, RBDA7-S, and Δ123A7-S, respectively (Fig. 1). RBDA7 and Δ123A7 contain 7 simultaneous Cys-to-Ala mutations at residues 452, 486, 569, 581, 585, 597, and 652 and are expressed exclusively as monomers (26, 27). Plasmids encoding each of the chimeric antigens were cotransfected together with a plasmid encoding DHBV S alone into 293-F cells to generate VLPs. In addition, control VLPs containing only the S antigen were produced in the same way by transfecting a plasmid encoding S alone. The tissue culture supernatants were collected and VLPs sedimented by ultracentrifugation through a 20% sucrose cushion. Expression of E2 and S was determined by SDS-PAGE and Western blotting (Fig. 2A). The results reveal that RBD-S, Δ123-S, RBDA7-S, and Δ123A7-S VLPs are comprised of two bands: a broad diffuse 50- to 75-kDa band migrating more slowly than soluble Δ123, indicative of the addition of the amino acid sequence of the 17 kDa S region, and a second band at 17 kDa corresponding to the expected molecular weight of S alone. Only the 17-kDa band was detected in VLPs produced by transfection of S alone. We verified that the 50- to 75-kDa bands contained E2 using bNMAb HCV1, specific to epitope I (also known as AS412 and domain E) encompassed by residues 412 to 423. As expected, both the 50- to 75-kDa band and the 17-kDa band were recognized by anti-S monoclonal antibody (MAb) 7C12. Higher yields of VLPs were recovered from the tissue culture fluids of cells transfected with the A7 variants, RBDA7-S and Δ123A7-S, than with their parental counterparts, suggesting that Cys-to-Ala mutations are more favorable to VLP formation. After confirming the expression of E2 and S, the VLPs were further purified on iodixanol gradients (Fig. 2B), and the resulting preparations were analyzed by SDS-PAGE and Western blotting to determine whether E2 and S cosedimented as VLPs. The fractions containing both E2 and S from the iodixanol gradient were combined, pelleted, and resuspended in phosphate-buffered saline (PBS) (Fig. 2C). The results strongly suggested that E2-S and DHBV S copurify in iodixanol gradients and are likely to represent VLPs. We found that the ratio of E2 to S differed between the Δ123A7-S and RBDA7-S preparations. Over the course of 4 consecutive preps, the ratio of Δ123A7-S to S ranged from a molar ratio of 1:2 to 1:4, with the average and most consistent (3 consecutive preps) being a 1:4 molar ratio. However, the molar ratio of RBDA7-S to S ranged from 1:4 to 1:10, with the average being a molar ratio of 1:7.

To examine the glycosylation status of E2 when fused to the S antigen and incorporated in VLPs when expressed in 293-F cells, deglycosylation was performed with either peptide-*N*-glycosidase F (PNGase F) or endoglycosidase H (Endo-H) (Fig. 2D). The results show that Δ123A7-S was sensitive to deglycosylation with PNGase F, reducing its molecular weight to the expected backbone size of 36 kDa. In contrast, Δ123A7-S was only modestly affected by Endo-H treatment, suggesting that it contains predominantly complex and hybrid glycans and a minimal amount of high-mannose carbohydrate. These results are comparable to those shown with the Δ123A7 monomer, indicating that the VLPs have a glycosylation pattern similar to that of the soluble protein expressed in 293-F cells. As expected, the 17-kDa band corresponding to DHBV S was not affected by any of the enzymes, consistent with a lack of N-linked carbohydrates.

To confirm VLP formation and surface presentation of epitopes, purified Δ123A7-S VLP samples were examined by transmission electron microscopy. Negative staining with uranyl acetate revealed spherical VLPs with diameters of approximately 60 nm (Fig. 2E). Immunoelectron microscopy demonstrated the presence of E2-S and S in individual VLP structures by bNMAb HC84.27, an epitope II (domain D, AS434)-directed antibody, and S-specific antibody 7C12, respectively (Fig. 2F). These data confirmed recombinant VLP formation and that the conformational domain D epitope recognized by HC84.27 was intact in the VLP-associated E2.

### E2-S VLPs display neutralizing epitopes and conceal nonneutralizing regions in E2.

To further characterize the antigenicity of the VLPs, an enzyme-linked immunosorbent assay (ELISA) was performed with a panel of well-characterized MAbs, including bNMAbs and nonneutralizing monoclonal antibodies (non-NMAbs) in a solid-phase assay. We previously compared the antigenicity of the soluble monomeric Δ123A7 protein to its parental Δ123 counterpart and high-molecular-weight versions thereof (28). In this study, we compared the antigenicity of Δ123A7 and RBDA7 with that of the Δ123A7-S and RBDA7-S version incorporated in VLPs.

**FIG 3:**
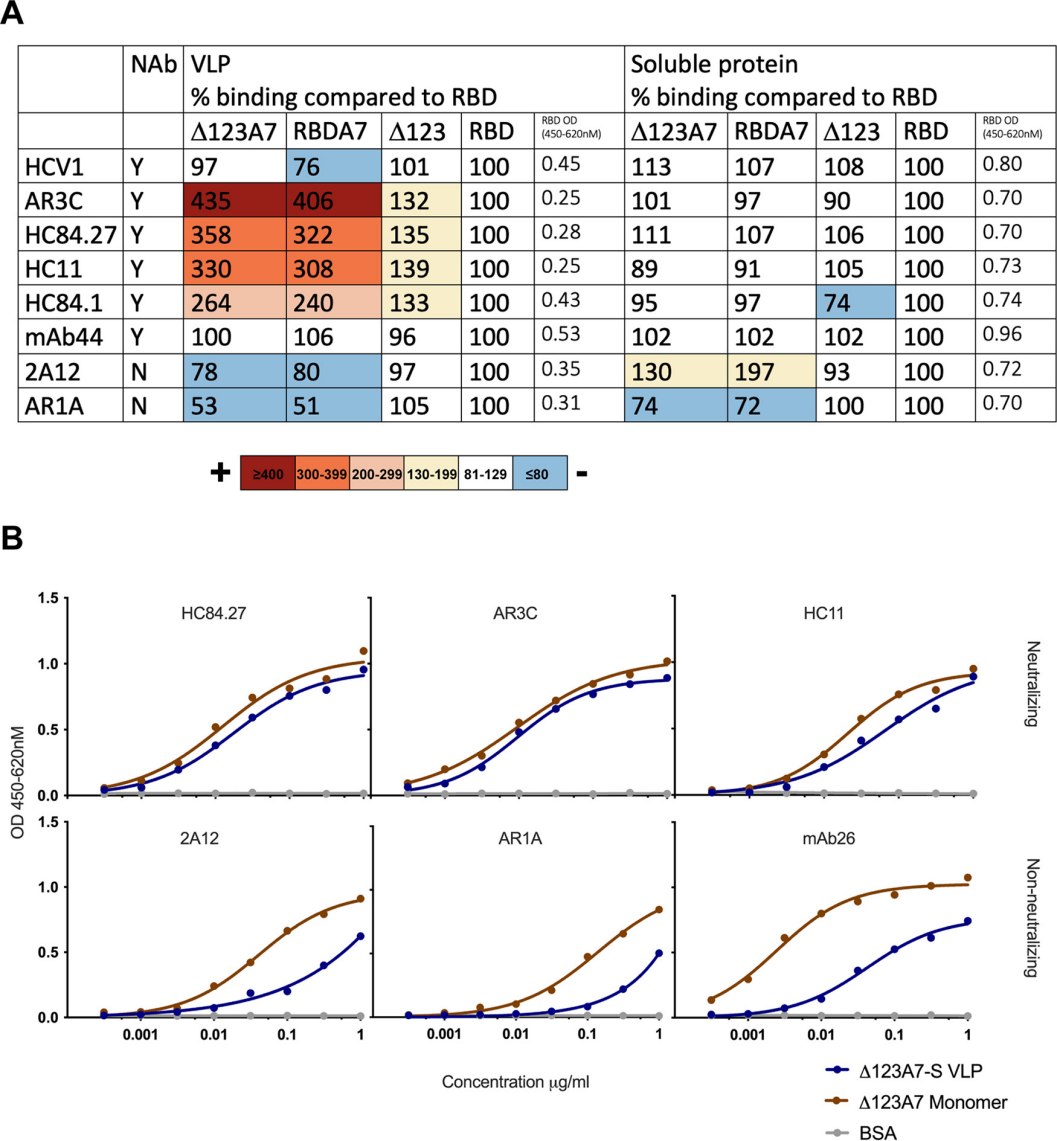
Antigenic characterization of E2-S VLPs and E2 soluble protein. (A) Binding of MAbs HCV1, AR3C, HC84.27, HC11, HC84.1, MAb44, 2A12, and AR1A to plate-bound chimeric E2-S VLPs (RBD-S, RBDA7-S, Δ123-S, or Δ123A7-S) at a single dilution of 0.5 μg/mL and soluble protein (RBD, RBDA7, Δ123, or Δ123A7) at a single dilution of 5 μg/ml. Specific binding was detected with the appropriate HRP-conjugated secondary antibody. MAb binding was calculated as a percentage compared to RBD binding, and mean percentages calculated from 4 separate experiments are shown. Binding: >400%, maroon; >300%, orange; >200%, light orange; >130%, yellow; and <80%, blue. Mean RBD binding absorbance values for VLP and soluble protein are indicated. (B) Comparison of the antigenicity of Δ123A7-S VLP and soluble monomeric Δ123A7 protein. Neutralizing MAbs HC84.27, AR3C, and HC11 and non-NMAbs 2A12, AR1A, and MAb26 were added to ELISA plates coated with either VLPs or soluble protein to assess reactivity. Specific binding was detected with the appropriate HRP-conjugated secondary antibody. Curves were fitted using nonlinear regression in GraphPad Prism.

VLPs displaying equivalent amounts of E2 or soluble versions of E2 protein were used to coat plates, and their degrees of binding to these antibodies were compared (Fig. 3A). This antibody panel included well-characterized antibodies that recognize discontinuous conformation-dependent epitopes and antibodies that recognize linear epitopes. MAb44 is directed to a linear epitope that is located within the CD81 binding loop (involving residues G523, P525, N540, W549, and Y613) and present in all constructs. This MAb bound each VLP form equivalently (Fig. 3A), confirming that similar amounts of E2 within VLPs were added to plates. The bNMAb HCV1 binds E2 via residues L413, N415, G418, W420, and I422 on the front face of E2 and showed a similar level of binding toward each of the E2-S VLP preparations, suggesting that this epitope was similarly exposed in all E2-containing VLP preparations.

To examine whether incorporation into VLPs affected the exposure of complex, conformation-dependent antibody epitopes, we examined the binding of bNMAbs AR3C, HC84.27, HC11, and HC84.1. These bNMAbs bound all forms of VLPs containing E2, indicating that these neutralizing epitopes were also preserved. The binding of bNMAbs to RBDA7-S and Δ123A7-S VLPs was enhanced compared to that to the RBD-S and Δ123-S VLPs, with AR3C displaying >4-fold enhancement, HC84.27 and HC11 3-fold enhancement, and HC84.1 >2-fold enhancement in binding (Fig. 3A). This contrasts with the similar levels of epitope exposure observed for the soluble version of these proteins compared to the RBD (Fig. 3A). In contrast, the non-NMAb AR1A displayed an almost 2-fold-lower level of binding toward RBDA7-S and Δ123A7-S VLPs than toward their parental counterparts, similar to the reduced binding observed for the soluble version of these E2 proteins. In the case of 2A12, the soluble version of RBDA7 and Δ123A7 displayed enhanced exposure of the epitope, while VLP forms showed reduced exposure.

A more detailed comparison of the antigenicity of Δ123A7-S VLPs, using concentrated preparations of VLPs and antibody titration, against the Δ123A7 soluble monomers was performed. This showed that the Δ123A7-S VLP and Δ123A7 monomers displayed similar reactivities toward bNMAbs HC84.27, AR3C, and HC11 (Fig. 3B), while the reactivity of Δ123A7-S VLPs toward non-NMAbs 2A12, AR1A, and MAb26 was reduced relative to its monomeric protein counterpart. Together, the data further support that neutralizing epitopes are maintained and exposed on VLPs incorporating A7 forms of E2.

### Characterization of the immune response to VLPs.

**FIG 4:**
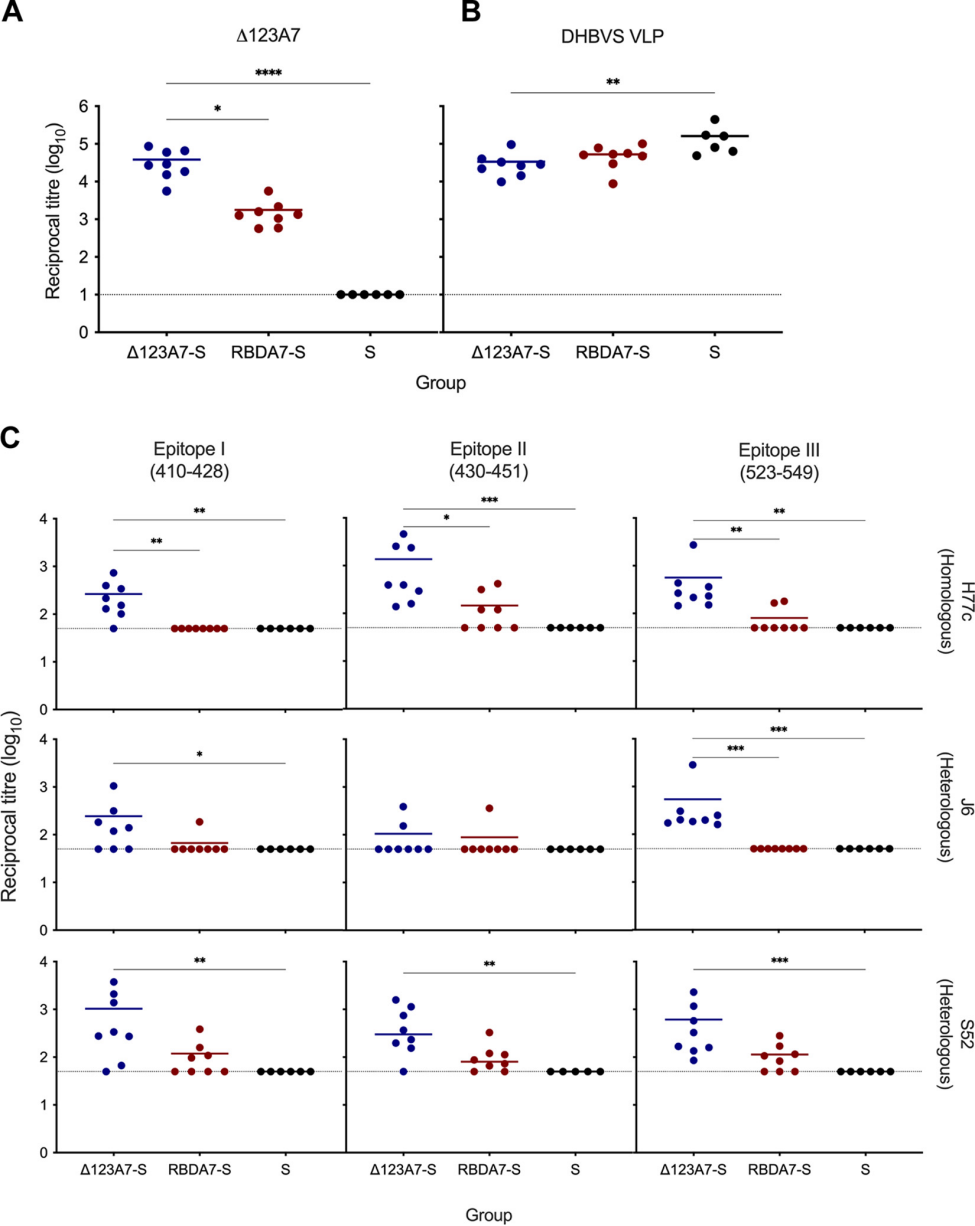
Reactivity of immune sera toward monomeric Δ123A7 protein (A) and S VLPs (B). Dilutions of immune sera were added to plates coated with monomeric Δ123A7 protein or S VLPs. Antibody titers were calculated as the reciprocal dilution of serum required for a 10-fold increase over binding to BSA (background binding). (C) Reactivity of immune sera toward continuous NAb epitopes. Homologous genotype 1a H77c peptides and heterologous genotype 2a J6 and genotype 3a S52 peptides (peptide 410-428, peptide 430-451, and peptide 523-549) were used to capture antibodies present in the immune serum. Antibody titers were calculated as the reciprocal dilution of serum required for a 10-fold increase over binding to BSA. The dotted line in panels A to C represents the lower limit of detection for the assay. Statistical analysis for panels A to C was performed using a Kruskal-Wallis test followed by Dunn’s multiple-comparison test in GraphPad Prism (v. 9). *, *P* ≤ 0.05; **, *P* ≤ 0.01; ***, *P* ≤ 0.001; ****, *P* ≤ 0.0001. The horizontal bars indicate the mean titers.

We have shown that Δ123A7-S and RBDA7-S efficiently form VLPs when coexpressed with DHBV S and expose key bNMAb epitopes. We therefore compared the immunogenicities of Δ123A7-S and RBDA7-S in guinea pigs. The VLP vaccine doses administered to guinea pigs were formulated to contain 10 μg of E2 or 40 μg of S alone, in combination with AddaVax adjuvant, 4 times at 3 weekly intervals. Sera obtained 2 weeks after the 3rd boost were examined for reactivity toward monomeric Δ123A7 and S VLPs by ELISA. Guinea pigs vaccinated with Δ123A7-S VLPs developed the highest titers of antibodies reactive to monomeric Δ123A7, with a mean titer of 10^4.5^ (Fig. 4A), and showed titers that were significantly higher than the titers generated in guinea pigs vaccinated with RBDA7-S VLPs (*P* < 0.05), which had a mean titer of 10^3.2^. All vaccine groups developed antibodies reactive toward VLPs containing S alone (Fig. 4B), with titers ranging from 10^4^ to 10^6^ and titers toward S being significantly higher in the S-alone group than in guinea pigs receiving Δ123A7-S VLPs (*P* < 0.01). No significant difference in reactivity to S was observed between the RBDA7-S group and the Δ123A7-S group (*P* > 0.05).

We next examined the specificity of antibodies present in the immune sera using biotinylated-E2 peptides corresponding to known linear HCV epitopes in ELISA (Fig. 4C). Peptide 410-428 (epitope I, also known as AS412, domain E) contains the epitopes for bNMAbs HCV1, MAb24, HC33.1, and AP33, including 2 important residues (W420 and H421) which are contact sites for CD81 (17, 29). Peptide 430-451 (epitope II, also known as AS432, domain D) contains CD81 contact residues N430, G436, W437, L438, G440, L441, F442, and Y443 and the epitope for bNMAbs HC84.1 and HC84.27 (17). Peptide 523-549 (epitope III) encompasses the CD81 binding loop and contains CD81 contact residues Y527, W529, G530, and D535 (30).

The results reveal that 7/8 guinea pigs vaccinated with Δ123A7-S VLPs generated binding titers ranging from 10^2^ to 10^3.6^ toward genotype 1a and 3a epitope I peptides and that 5/8 of these sera cross-reacted with the genotype 2a epitope I peptide sequence (Fig. 4C). In contrast, guinea pigs vaccinated with RBDA7-S VLPs failed to generate homologous epitope I-specific antibodies, while 1/8 and 4/8 of these guinea pigs generated cross-reactive antibodies to genotype 2a and 3a peptides, respectively. All guinea pigs vaccinated with Δ123A7-S VLPs and 4/8 guinea pigs vaccinated with RBDA7-S VLPs generated antibodies reactive to genotype 1a epitope II peptide, with mean titers of 10^3.2^ and 10^2.3^, respectively. Totals of 2/8 guinea pigs vaccinated with Δ123A7-S VLPs and 1/8 guinea pigs vaccinated with RBDA7-S VLPs showed cross-reactivity with genotype 2a epitope II, with a mean titer of 10^2^. Totals of 7/8 Δ123A7-S guinea pigs and 6/8 RBDA7-S guinea pigs reacted to genotype 3a epitope II peptide, with mean titers of 10^2.5^ and 10^2^, respectively. In the case of epitope III, all guinea pigs in the Δ123A7-S group generated genotype 1a, 2a, and 3a epitope-reactive antibodies, with binding titers ranging from 10^2^ to 10^3.5^ and a mean binding titer of 10^2.7^. In contrast, only 2/8 guinea pigs in the RBDA7-S group produced genotype 1a epitope III-reactive antibodies, and no animals in this group produced genotype 2a epitope III-reactive antibodies, whereas 5/8 had reactivity to genotype 3a epitope III peptide.

**FIG 5:**
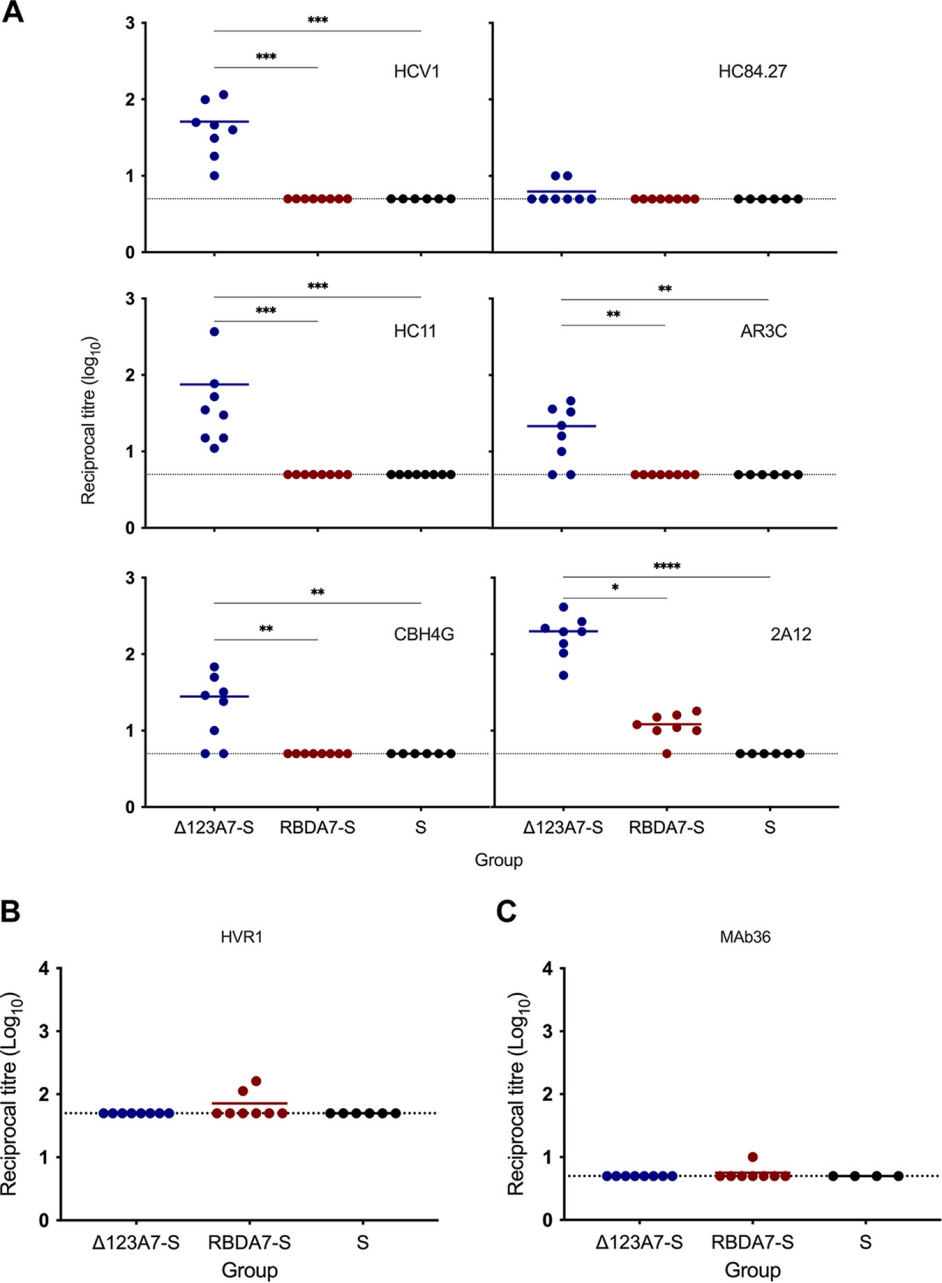
Analysis of antibody specificity. (A) Competitive ELISA to determine specificity of the antibody response. Serial dilutions of immune sera were mixed with a constant amount of MAb prior to incubation with plate-bound Δ123. The reciprocal dilutions of serum required for 50% inhibition (ID_50_) relative to MAb binding in the absence of sera are shown. (B) Reactivity of serum to HVR1 peptide. Plate-bound genotype 1a H77c HVR1 peptide was used to capture antibodies present in the immune serum. Antibody titers were calculated as the reciprocal dilution of serum required for a 10-fold increase over background binding. (C) Competitive ELISA to determine specificity of the antibody response to HVR1 MAb36. Serial dilutions of immune serum were added to a constant amount of MAb36 prior to incubation with plate-bound Δ123. The reciprocal dilution of serum required for 50% inhibition was calculated using binding of MAb in the absence of serum as 100% binding. The dotted line in panels A to C represents the lower limit of detection for the assay. Statistical analysis for panels A to C was performed using a Kruskal-Wallis test followed by Dunn’s multiple-comparison test in GraphPad Prism (v. 9). *, *P* ≤ 0.05; **, *P* ≤ 0.01; ***, *P* ≤ 0.001; ****, *P* ≤ 0.0001. The horizontal bars indicate the mean titers.

We next investigated the ability of the sera to compete with the bNMAbs HCV1, HC84.27, HC11, and AR3C and the non-NMAbs CBH4G and 2A12 for binding to soluble E2 D123 antigen in a competition ELISA (Fig. 5A). The results show that all guinea pigs vaccinated with Δ123A7-S produced antibodies that blocked the binding of HC11 and HCV1 to the E2 D123; however, immunization with RBDA7-S failed to generate HC11- and HCV1-like specificities (*P* = 0.001). Furthermore, 6/8 Δ123A7-S-vaccinated guinea pig sera competed with AR3C for binding to E2 D123, whereas none of the guinea pigs in the RBDA7-S group generated AR3C-like specificities (*P* = 0.001). Significant HC84.27 blocking specificities were not elicited by either antigen, consistent with an absence of domain D-directed antibodies.

In the case of nonneutralizing domains, 6/8 guinea pig sera within the Δ123A7-S immune group had CBH-4G-like specificities, and all had 2A12-like specificities, with significantly higher titers than the RBDA7-S group (*P* = 0.05) (Fig. 5A). Taken together, these results suggest that Δ123A7-S VLPs produced antibodies that overlap the epitopes recognized by HCV1, HC11, and AR3C and are thus a superior antigen to RBDA7-S VLPs. However, Δ123A7-S also generated antibodies targeting the nonneutralizing face of E2.

To determine whether RBDA7-S VLPs elicited HVR1-directed antibodies, a direct HVR1 peptide binding ELISA was employed (Fig. 5B). A total of 2/8 guinea pig sera from the RBDA7-S group showed weak reactivity to HVR1 peptide, indicating that HVR1 is not highly immunogenic in the RBDA7-S VLPs. To support this result, we also determined the ability of the serum to compete for HVR1 binding (in the context of the intact RBD) with HVR1-specific MAb36 (17) (Fig. 5C). A total of 1/8 sera of guinea pigs vaccinated with RBDA7-S competed weakly with this antibody. As expected, Δ123A7-S VLPs, which do not include the HVR1, did not elicit antibodies binding to HVR1 peptide or competing with MAb36.

**FIG 6:**
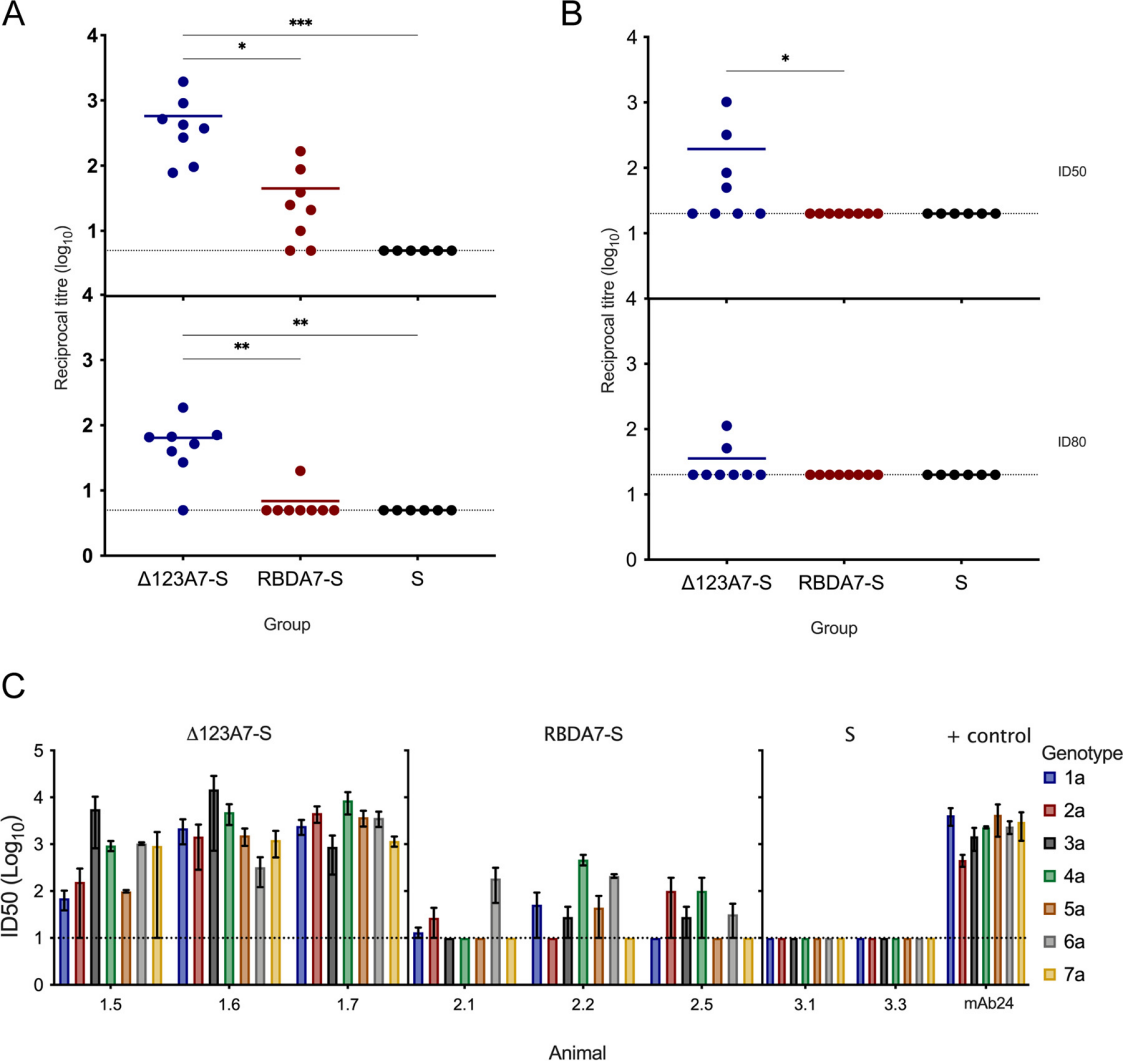
(A) Ability of immune sera to inhibit the binding of homologous H77c RBD to CD81-LEL. Immune sera were precleared of interfering antibodies and incubated with H77c RBD before being added to CD81-LEL-coated plates. RBD binding was detected by E2-specific MAb H53 and HRP-conjugated secondary antibody, and the reciprocal dilution of serum required to inhibit E2-CD81 interaction by 50% and 80% was calculated. (B) Ability of immune serum to neutralize homologous genotype 1a pp virus. Serial dilutions of Δ123A7-S VLP, RBDA7-S VLP, or S VLP precleared sera were mixed with HCVpp (H77c, genotype 1a). Luciferase activity was measured, and the reciprocal dilution of serum required to achieve 50% and 80% neutralization was calculated. Statistical analysis for panels A and B was performed using a Kruskal-Wallis test followed by Dunn’s multiple-comparison test in GraphPad Prism (v. 9). *, *P* ≤ 0.05; **, *P* ≤ 0.01; ***, *P* ≤ 0.001. The horizontal bars indicate the mean titers. (C) Ability of sera to neutralize diverse HCV genotypes. Serial dilutions of precleared Δ123A7-S VLP, RBDA7-S VLP, or S VLP sera or MAb24 miniperm (7 μg/mL) were mixed with either HCVpp (genotype 1a) or HCVcc (genotypes 2 to 7). Luciferase activity was measured, and the reciprocal dilution of serum required to achieve 50% neutralization was calculated. The data were derived from three independent experiments performed in triplicate, with bars representing the means and error bars showing SEM. In panels A to C, the dotted line represents the lower limit of detection of the assay.

To determine if the VLP immune sera possessed CD81-E2-blocking antibodies, we used a previously described ELISA employing plates coated with a recombinant form of the large extracellular loop of CD81 and solution-phase E2 RBD (19). The initial results showed that there was a high level of background CD81 inhibition unrelated to E2 antigen, suggesting that the guinea pigs may have generated antibodies toward contaminating CD81 present in the VLP preparation. To remove potentially cross-reactive non-E2-specific antibodies, the serum was precleared of interfering antibodies on 293-F cells prior to analysis. Precleared immune sera from the Δ123A7-S-vaccinated guinea pigs possessed significantly higher (*P* = 0.001) titers of CD81-E2-blocking antibody relative to RBDA7-S VLP immune sera (mean 50% inhibitory dose [ID_50_], 10^2.8^ versus 10^1.5^, respectively; mean ID_80_, 10^1.7^ versus 10^0.6^, respectively) (Fig. 6A).

### E2-S VLPs generate broadly neutralizing antibodies.

The ability of immune serum to neutralize homologous genotype 1a H77c pseudotyped particle (pp) virus was examined using precleared immune serum. Development of NAbs was present but inconsistent, with 4 of 8 guinea pigs immunized with Δ123A7-S generating NAbs to genotype 1a pseudotyped viruses, the mean ID_50_ of this group being 10^2.3^ (Fig. 6B). In contrast, RBDA7-S did not elicit homologous neutralizing activity.

Three guinea pigs from each of the groups receiving Δ123A7-S and RBDA7-S with the highest titers of CD81-E2-blocking and genotype 1a-neutralizing antibodies were analyzed for their ability to neutralize diverse HCV genotypes using cell culture-derived viruses (Fig. 6C). Sera from all three guinea pigs examined in the Δ123A7-S group (animals 1.5, 1.6, and 1.7) were able to neutralize HCV genotypes 1a, 3a, 4a, 5a, and 6a, and 2/3 sera were able to neutralize genotype 2a and 7a cell culture-derived viruses (HCVcc). Lower levels of cross-neutralization (genotype 2a, 3a, 4a, 5a, and 6a viruses) were observed with 2/3 sera from the RBDA7-S immune group (animals 2.2 and 2.5), with the ID_50_ being at least an order of magnitude lower than those obtained with Δ123A7-S immune sera.

**FIG 7:**
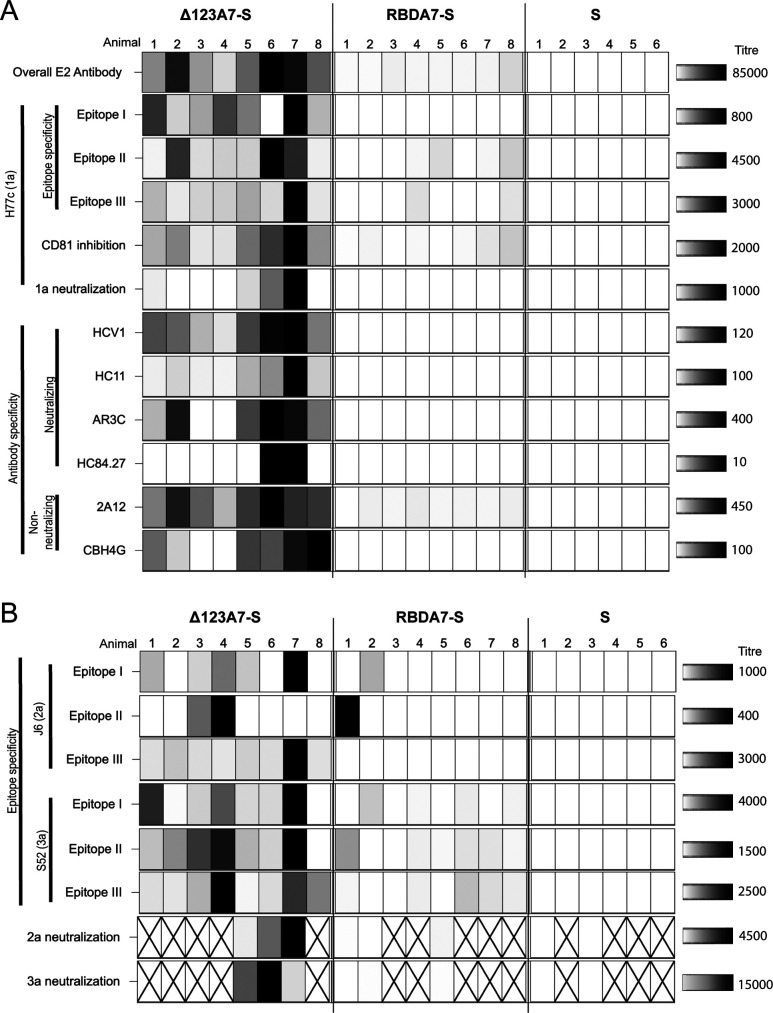
Binding and neutralizing titers for individual animals vaccinated with either Δ123A7-S VLPs, RBDA7-S VLPs, or S VLPs. Titers generated against homologous genotype 1a (H77c) (A) and heterologous genotype 2a (J6) and genotype 3a (S52) (B) were plotted in a heat map. Higher titers are represented by increasingly darker tone. A white box indicates activity below the level of detection for that assay. An “X” indicates that the experiment was not conducted.

While there were insufficient numbers of animals to perform a meaningful correlation analysis, we examined the combined data to observe trends associated with homologous and heterologous neutralization (Fig. 7). Animals 6 and 7 from the Δ123A7-S group produced the highest homologous and G2a and G3a cross-neutralization, respectively (Fig. 7A). This was associated with the highest overall E2 antibody titers and E2-CD81-blocking antibodies and antibodies that overlap bNMAbs that recognize domains D, E, and AR3. These animals also produced high titers of antibodies that targeted nonneutralizing epitopes (2A12 and CBH4G). The strong G2a cross-neutralization observed for animal 7 was also associated with high titers of antibodies toward G2a-epitopes I and III. However, the strong G3a cross-neutralization observed for animal 6 was not associated with cross-reactive antibody specificities to any of the synthetic peptides to epitope I, II, or III. Previous studies using Δ123 as an immunogen in guinea pigs showed similar associations with domain D, domain E, and AR3 specificities (19).

## DISCUSSION

One of the challenges for developing an HCV vaccine is the need to generate a protective immune response toward the 8 genetically and antigenically distinct genotypes (1, 31). Human vaccination studies to date have failed to elicit strong and broad neutralization activity against HCV (32–36). Previously, we found that the oligomeric state of recombinant E2 was critical to the generation of bNAbs in guinea pigs. A disulfide-linked HMW form of E2Δ123 was a more potent immunogen than the monomeric form and was able to elicit antibodies with broad neutralizing activity against 7 genotypes of HCV (19). We suggested that this was due to the occlusion of nonneutralizing epitopes within the HMW form of E2, which redirects the immune response to the neutralizing epitopes. Drawbacks for vaccine development of the HMW form of E2 included low yield and difficulties in purification.

In the current study, we sought to develop a more readily scalable multivalent immunogen by engineering E2 nanoparticle VLPs using the DHBV S system. Our studies indicate that E2-S VLPs can be efficiently formed by genetically fusing the E2 glycoprotein and DHBV S antigen. A combination of analyses by electron microscopy, Western blotting, and ELISA showed that VLPs formed spherical structures containing both E2-S and S presented on the surface of the particle. As the glycosylation of E2 is an important determinant in folding and antigenicity, we confirmed that E2-S contains N-linked complex/hybrid glycans when expressed in mammalian cells. We found that Δ123A7-S incorporated more E2 onto each particle than RBDA7-S, suggesting that the presence of the hypervariable regions adversely impacts assembly. We also found that the A7 forms of E2 were incorporated into VLPs at significantly higher levels than the parental forms. This may be because the A7 forms are likely to have a simplified folding pathway and therefore assemble more efficiently as VLPs. The A7 forms of E2 may also have an intrinsically more favorable conformation for VLP assembly and stability. The parental forms of E2 have at least one free cysteine that could form intermolecular disulfide bonds with cysteine residues within other E2 molecules or the DHBV S polypeptide and disrupt VLP formation. Consistent with the adoption of a more native-like conformation, RBDA7-S and Δ123A7-S VLPs were 3 to 4 times more reactive to conformational but not nonconformational bNMAbs than the non-A7 forms. The AR3C epitope and the HC84.27 epitope overlap the binding site for CD81, and the residues constituting the epitopes are 80 to 100% conserved across all HCV isolates (37, 38). The enhanced reactivity of RBDA7-S and Δ123A7-S VLPs with these antibodies therefore shows their immunogenic potential for eliciting bNAbs effective across HCV genotypes. In contrast, the reactivity of the A7 forms to nonneutralizing antibodies AR1A, 2A12, and MAb26 was reduced, indicating at least partial occlusion of the nonneutralizing face of E2 in VLPs.

When used to immunized guinea pigs, the Δ123A7-S VLPs generated higher titers of E2-CD81-blocking antibodies and cross-reactive epitope I/AS412/domain E antibodies than RBDA7-S VLPs. In addition, Δ123A7-S produced higher titers of HCV1, HC11, and AR3C blocking antibodies than the RBDA7-S VLPs. The production of these antibody specificities correlated with 4/8 Δ123A7-S VLP-immunized animals showing antibodies capable of neutralizing homologous HCV and 3 of these animals generating bNAbs to all 7 major genotypes of HCV. Additionally, these 3 animals showed ID_50_ values close to those previously observed using 100 μg of the HMW form of Δ123 (19). These results are consistent with occlusion of the nonneutralizing face of E2 and redirection of the immune response to the neutralizing face. Competition assays using immune sera generated with Δ123A7-S VLPs showed significant titers of antibodies able to block the binding of the non-NMAb 2A12 and CBH4G antibodies to E2, indicating that parts of the nonneutralizing face or adjacent regions are accessible to some degree after immunization.

Previous HCV vaccine studies using 30 to 200 μg of E2 immunogens in mice, guinea pigs, and nonhuman primates resulted in high titers of NAbs (19, 39–42). In the current study, the VLPs contained the equivalent of 10 μg of E2 and were administered using the adjuvant Addavax. While only 4/8 animals generated NAbs, and only 3 generated bNAbs against all 7 HCV genotypes, these data suggest that VLP delivery of HCV E2 antigens may have some dose-sparing advantages over the use of soluble immunogens. This may relate to the repetitive antigen display and large size, which is optimal for recognition by dendritic cells (22). However, future studies should examine alternative doses and adjuvants to ensure that all animals vaccinated generate NAbs, as observed by Vietheer et al. (19). VLP-associated antigens can be presented to major histocompatibility complex (MHC) class I and II molecules to activate and stimulate CD8^+^ T cells and CD4^+^ T helper cells after uptake by dendritic cells (43–46). HCV-specific T cells play a critical role in viral control and clearance of HCV, with CD4^+^ and CD8^+^ T cells targeting different viral antigens (47, 48). The production of interferon gamma by CD8^+^ T cells induces antiviral effects and inhibits replication (49, 50), and CD4^+^ T cells activate antigen-presenting cells and provide help for CD8^+^ T cells by cytokine production (51). We did not investigate whether E2-S VLPs induce T cell responses due to the limited availability of reagents for such work in guinea pigs. Future vaccine trials conducted with vaccinated mice and humans could usefully examine if T cell responses contribute to the quality and magnitude of the humoral response generated by E2-S VLP vaccines.

Ideally, a low-cost vaccine is required to meet global health needs. Studies have demonstrated that DHBV S VLPs can be assembled and expressed in the methylotrophic yeast Hansenula polymorpha when chimerized at the N terminus with the antigens from 4 different animal viruses (25). These VLPs were shown to have a homogenous structure, with surface exposure of the foreign antigens and colocalization of both S and foreign antigen as shown by immunoelectron microscopy and superresolution microscopy. Expression in yeast systems is likely to yield a dramatic reduction in cost of manufacture but will require a reevaluation of the antigenicity and glycosylation status of the VLPs.

In conclusion, a vaccine to prevent HCV infection is an achievable goal and the critical addition to the elements required to reach elimination targets. We have developed a VLP vaccine candidate that can generate broad and robust neutralization of HCV with the potential for scalability and reduced production costs.

## MATERIALS AND METHODS

### Production of E2-S plasmids.

E2-S plasmids were cloned using an overlap PCR strategy. Plasmids encoding sequences of Δ123A7, RBD, and RBDA7 were used as the template for reaction mixtures containing the forward primer 5′-E2/NheI (5′-ACCGCTAGCGCCACCATGAACCCCCTGCTGATCC-3′), encoding the NheI restriction site (underlined), and reverse primer 3′-E2/KpnI (5′-CGAAGGTACCCTCGCTGCGGTCCCGATCTTCCAGG-3′), encoding the KpnI restriction site (underlined). The DHBV S fragment was amplified from pCI-LS (52) with forward primer 5′-DHBVS/KpnI/Overlap (5′-GACCGCAGCGAGGGTACCTTCGGGGGAATACTAGCTGG-3′), encoding the KpnI restriction site (underlined), and reverse primer 3′-DHBVS/Stop/XhoI (5′-GGCCTCGAGCTAACTCTTGTAAAAAAGAGCAGACAGCGTG-3′), encoding XhoI the restriction site (underlined). PCR was performed using the Expand high-fidelity PCR system (Roche, Mannheim, Germany). Using the first-round product of Δ123, overlap PCR was performed using the external primers followed by digestion with restriction enzymes NheI-HF and XhoI. The DNA fragments were then ligated into the NheI and XhoI sites of pcDNA3 using T4 DNA ligase (Invitrogen) according to the manufacturer’s instructions to create Δ123-S. To produce the RBD-S, RBDA7-S, and Δ123A7-S plasmids, Δ123-S was digested with NheI-HF and KpnI to remove Δ123 and then replaced with similarly digested first-round PCR products of Δ123A7, RBD, and RBDA7 by ligation. pcDNA3-E2-S constructs were validated by restriction enzyme digestion and subsequent sequencing using BigDye Terminator v3.1 sequencing kit (Applied Biosystems).

### Soluble protein production and monoclonal antibodies.

Soluble E2 proteins were produced via transient transfection of Freestyle 293-F cells. Plasmids pE2RBD, pE2RBDA7, pE2Δ123, and pE2Δ123A7 were transfected using 293Fectin (Invitrogen) according to the manufacturer’s instructions. Soluble glycoproteins secreted into the culture supernatants were affinity purified using cobalt-charged TALON metal affinity resin (Clontech) and then subjected to size exclusion chromatography on a Superdex 200 prep-grade 16/600 column (GE Healthcare) using the ÄKTA pure fast protein liquid chromatography (FPLC) system (GE Healthcare) equilibrated in PBS (pH 6.8). Monomeric species, as previously defined (19), were pooled and concentrated through a centrifugal filter with a nominal molecular weight cutoff (30 kDa; Amicon).

Anti-S MAb 7C12 was kindly provided by ARTES Biotechnology (Langenfeld, Germany). Synthetic MAbs specific to epitopes located within E2 (HCV1, AR3C, 2A12, and HC84.27) were expressed and purified from 293-F cells as previously described (53). E2 MAb24 and MAb44 were produced from mouse hybridoma cell lines as previously described (17). E2 MAb AR1A was kindly provided by Mansun Law (Scripps Research, CA).

### VLP production and purification.

Virus-like particles were produced by transiently cotransfecting 293-F cells with the E2-DHBV S and DHBV S plasmids at a ratio of 1:4 using 293fectin transfection reagent (Invitrogen). DHBV S VLPs were produced by transient transfection of 293-F cells with DHBV S plasmid and in some cases using a stable transfected cell clone. Cultures were incubated for 5 days, and cell culture supernatants containing secreted VLPs were harvested and filtered (0.45 μm). Purification of VLPs was performed by ultracentrifugation at 100,000 × *g* at 4°C on a 4-mL 20% (wt/vol) sucrose cushion for 2 h using an SW 28 Ti swinging-bucket rotor in an Optima L-100 XP ultracentrifuge (Beckman Coulter), and the resulting pellet containing VLPs was resuspended in 200 μL of PBS (pH 6.8). The VLPs were overlaid onto a 5 to 40% (wt/vol) OptiPrep (Merck) gradient, followed by further ultracentrifugation at 100,000 × *g* at 4°C for 4 h as described above, and 1-mL fractions were collected. Each fraction was analyzed by Western blotting, probing with anti-E2 (HCV1) and anti-S (7C12) antibodies. Fractions containing both E2-S and S proteins were buffer exchanged into PBS (pH 6.8) using a centrifugal filter with a nominal molecular weight cutoff (30 kDa; Amicon).

### SDS-PAGE and Western blotting.

Samples were denatured and reduced using standard conditions in the presence of 3% (vol/vol) beta-mercaptoethanol and separated on 12% (wt/vol) polyacrylamide gels using a Tris-glycine-SDS buffer system (0.025 M Tris [pH 8.3], 0.19 M glycine, 0.1% [wt/vol] SDS). Proteins were transferred onto a nitrocellulose membrane using a Tris-glycine transfer buffer (0.025 M Tris [pH 8.3], 0.19 M glycine, 20% [vol/vol] methanol). Membranes were blocked with 5% skim milk in PBS and probed with antibodies directed toward E2 (HCV1) or S (7C12), followed by detection with fluorescently conjugated secondary antibodies specific for the primary antibody species. Primary and secondary antibodies were diluted in PBS containing 5% skim milk. Membranes were imaged on an Odyssey infrared imaging system (Li-COR Biosciences). The amount and proportion of E2 and S within VLPs were quantified using Image Studio software (LI-COR Biosciences). To estimate the quantity of VLPs produced, the amount of E2 within each VLP preparation was quantitated against a known amount of purified E2.

### Glycan analysis.

The pattern of N-linked glycosylation of VLP-associated E2 was determined by treatment with PNGase F (Sigma) or Endo-H (Sigma). Samples containing 1 μg of protein were first denatured in 10 μL of buffer containing 0.5% (wt/vol) SDS and 40 mM dithiothreitol (DTT), with heating to 95°C for 10 min. High-mannose glycans were specifically removed using 1 unit of Endo-H in 50 μM sodium acetate (pH 6). All N-linked glycans were removed using 1 unit of PNGase F in 50 μM sodium phosphate (pH 7.5), containing 1% (vol/vol) NP-40. Untreated samples were also prepared in the absence of enzyme. Samples were then incubated at 37°C for 4 h before separation and detection via SDS-PAGE and Western blotting as described above.

### Negative-staining electron microscopy.

Five microliters of purified E2Δ123A7-S VLPs was incubated on the top of a glow-discharged carbon-coated 200-mesh copper grid (Electron Microscopy Services) for 1 min at room temperature before blotting with filter paper. The grid was briefly rinsed three times in drops of ultrapure water and blotted after each rinse. The grid was then incubated sample side down on a drop of 1% (wt/vol) uranyl acetate for 10 s, before blotting and air drying for 10 min at room temperature. The grid was then imaged on a 120 keV Tecnai Spirit G2 transmission electron microscope equipped with a 4K Eagle camera (FEI).

### Immunoelectron microscopy.

Five microliters of purified E2Δ123A7-S VLPs (1/200 dilution in PBS) was incubated on the surface of a glow-discharged continuous 200-mesh carbon grid for 3 min at room temperature before blotting and rinsing with PBS containing 1% (wt/vol) bovine serum albumin (BSA) and 0.005% (vol/vol) Tween 20 for 3 min. The grid then underwent a series of 30-min incubations with primary and secondary antibodies diluted in PBS containing 5% (wt/vol) BSA and 0.05% (vol/vol) Tween 20: (i) 100 ng of E2-specific human MAb HC84.27, (ii) 100 ng of S-specific mouse MAb 7C12, (iii) 4 μg of 5-nm gold-conjugated anti-human immunoglobulin, and (iv) 4 μg of 10-nm gold-conjugated anti-mouse immunoglobulin. The grid was rinsed twice in PBS containing 1% (wt/vol) BSA and 0.005% (vol/vol) Tween 20 and blotted between incubations. Finally, the grid was rinsed with ultrapure water and blotted. The grid was then incubated sample side down on a drop of 2% (wt/vol) uranyl acetate for 30 s, before blotting and air drying for an hour at room temperature. Imaging was performed on a 120 keV JIB-1400FLASH transmission electron microscope equipped with a Matataki Flash sCMOS camera (JEOL Ltd.).

### Animal vaccinations.

Tricolor guinea pigs were vaccinated subcutaneously with either Δ123A7-S VLPs or RBDA7-S VLPs (8 per group) containing the equivalent of 10 μg of E2 or vaccinated with VLPs containing S alone (6 per group) containing the equivalent of 40 μg of S antigen, formulated with AddaVax (InvivoGen, San Diego, CA) adjuvant at a 1:1 ratio in a total volume of 200 μL. All guinea pigs received four doses of the vaccine formulation at weeks 0, 3, 6, and 9. At week 11, blood was collected via nonrecovery cardiac puncture. This was performed under anesthesia with isoflurane/oxygen (induction, 3 to 4%; maintenance, 1 to 2%; 1.2 L/min). Guinea pigs were then humanely euthanized by intracardiac injection of potassium chloride (KCl; 4 M; 2.5 mL/kg) under anesthetic (isoflurane). Guinea pigs used for immunizations were cared for in accordance with the Australian code for the care and use of animals for scientific purposes (59), under SAHMRI AEC project number SAM210.

### ELISA.

The reactivity of MAbs or serum to VLPs or E2 was tested by enzyme-linked immunosorbent assays (ELISA) using Maxisorp flat-bottom 96-well plates (Nunc). Plates were coated with either 0.5 or 1 μg/mL of E2-containing VLPs or 1 or 5 μg/mL of soluble recombinant monomeric E2 proteins in soluble form in 50 mM carbonate-bicarbonate buffer (pH 9.6) and incubated overnight at 4°C as previously described (28). The reactivity of serum to E2 peptides was tested by ELISA using plate-bound avidin to capture biotinylated peptides as previously described (28). Sera or MAbs were added at a 0.5 log_10_ dilution and bound antibody was detected with horseradish peroxidase (HRP)-conjugated secondary antibody. The ability of antibodies within immune sera to compete with MAbs for binding to monomeric RBD was measured as previously described (28). Inhibitory titers were expressed as the reciprocal dilution of immune serum that reduces the binding reaction being competed by 50% (ID_50_) where binding in the absence of sera is equivalent to 100% binding.

### CD81 inhibition assay.

The capacity for immune serum to inhibit E2 binding to the CD81 binding site was measured using a previously described E2-CD81 inhibition of binding assay (19) except that immune sera were precleared of antibodies reactive with contaminating 293-F cellular proteins. Serum was incubated with 20 million viable 293-F cells per 125 μL of serum on ice for 2 h. This method was repeated 10 times to total 200 million cells per 125 μL of serum.

### Neutralization assays.

Retroviral pseudotyped particles containing E1 and E2 (HCVpp) were produced by cotransfecting HEK293T cells with pE1E2 and the HIV-1 NL4-3 ΔEnv Vpr luciferase reporter vector (pNL4-3.Luc.R-E-) (54–56). After 72 h, tissue culture medium was filtered (0.45 μm) and used immediately in neutralization assays. For the production of cell culture-derived viruses (HCVcc), full-length *in vitro*-transcribed RNA from 2a (J6)-RLuc Δ40, 3a (S52)-RLuc Δ40, 4a (ED43)-RLuc Δ40, 5a (SA13)-RLuc Δ40, 6a (HK6a)-RLuc Δ40, and 7a (QC69)-Rluc Δ40 (kind gifts from Jens Bukh) (57) were electroporated into Huh 7.5 cells as described previously (58). After 72 h, tissue culture fluid containing HCVcc was collected and filtered (0.45 μm). Immune serum that was precleared as described above was then added to either HCVpp or HCVcc, and neutralization assays were performed as described previously (19). The neutralization capacity of the immune sera was tested using luciferase reporter assays and HCVpp or HCVcc as previously described (19). The reciprocal dilution of serum required to achieve 50% inhibition of virus entry was used to calculate the ID_50_ from three independent experiments.

### Statistical analysis.

Experiments were performed at least three times except where indicated. All data were plotted in GraphPad Prism (v. 9). Means ± standard deviations (SD) or standard errors (SE) as indicated are shown. Nonlinear regression was performed using the Hill slope equation, and the interpolation function was used to derive titers. Data were statistically compared using the nonparametric Kruskal-Wallis test with Dunn’s multiple comparisons.

### Data availability.

Data will be shared upon request to Heidi E. Drummer.
